# Supplementary material for: Association of the platelet-to-high-density lipoprotein cholesterol ratio (PHR) with metabolic syndrome and metabolic overweight/obesity phenotypes: A study based on the Dryad database
Source: PLoS One. 2025 May 6;20(5):e0321625. doi: 10.1371/journal.pone.0321625 (PMC12054858; doi:10.1371/journal.pone.0321625)
Supplement: Attachment — Submitted filename: PHR.docx [file pone.0321625.s001.docx]

Dear This retrospective study assess the PHR in normal built, overweight, and obese people with / without metabolic disturbance. The following comments required explanation: 1: Typing errors e.g. reference 2: The ATP III is the criteria of metabolic syndrome. Why this scientific term is not used? 3: NAFLD is an old term. It is substituted with metabolic dysfunction associated with liver disease 4: The diagnostic criteria of body mass index is not according to the WHO criteria. Please add a reference of using the criteria of BMI that appeared in the article Regards
