# Supplementary material for: Association of the platelet-to-high-density lipoprotein cholesterol ratio (PHR) with metabolic syndrome and metabolic overweight/obesity phenotypes: A study based on the Dryad database
Source: PLoS One. 2025 May 6;20(5):e0321625. doi: 10.1371/journal.pone.0321625 (PMC12054858; doi:10.1371/journal.pone.0321625)
Supplement: Attachment — Submitted filename: Response to Reviewers - 2025-03-10T091131.124.docx [file pone.0321625.s002.docx]

**Dear Academic Editor**

On behalf of my co-authors, we thank you very much for giving us an opportunity to revise our manuscript. We appreciate editor and reviewers very much for their positive and constructive comments and suggestions on our manuscript entitled “**Platelet-to-high-density lipoprotein cholesterol ratio and risk of metabolically unhealthy phenotype: A study based on the Dryad database**” (Manuscript Number: **PONE-D-25-00775**).

We modified the manuscript format in line with the style requirements of PLOS ONE. We uploaded the results to the figshare common repository (doi: 10.6084 / m9. Figshare. 28498460). I possess the ORCID iD and it has been verified in Editorial Manager. We have meticulously checked the references to guarantee their completeness and correctness.

Thank you for your detailed instructions and suggestions on the data availability statement! We have carefully read and understood the requirements of your open Data policy. We will immediately review and update our data availability statement to ensure compliance with the journal's policies. Data Availability statement: “Data are available in a public, open access repository. The datasets analyzed in this study are openly available in the Dryad Digital Repository ([https://doi.org/10.5061/dryad.7d7wm3809](https://doi.org/10.5061/dryad.7d7wm3809)).”

We have relocated the ethics statement to the Methods section and removed it from other sections to ensure compliance with the journal's requirements.

We have studied reviewer’s comments carefully and have made revision which marked in red in the paper. We have tried our best to revise our manuscript according to the comments.Please find attached the revised version, which we respectfully submit for your kind consideration.

We would like to express our great appreciation to you and reviewers for comments on our paper. Looking forward to hearing from you.

Thank you and best regards.
Yours sincerely,

Corresponding author:
Name: Ping Zhang

E-mail:1749743784@qq.com

**Responses to the Editor’s comments**

**Comment 1: Typing errors e.g. reference**

**Response:** Thank you for bringing the typing errors to my attention. I have thoroughly reviewed the manuscript and corrected all identified errors. Your feedback is greatly appreciated, and I strive to ensure the accuracy and clarity of the manuscript.

**Comment 2: The ATP III is the criteria of metabolic syndrome. Why this scientific term is not used?**

**Response:** Thank you for your review of our paper and your valuable comments.The diagnostic criteria for metabolic syndrome define metabolic abnormalities through a combination of indicators rather than as a single disease. This classification aligns better with its pathological complexity, dynamic evolution, and clinical intervention needs. In the context of obesity, the concept of metabolic health has garnered significant attention in the scientific community. The concept of metabolic health is not exclusive to obesity; it is increasingly used to assess the risk of cardiovascular disease and diabetes-related complications[1,2].

The predominant approach in research defines metabolic health as the absence of metabolic syndrome. For example, a 2019 systematic review identified 35 cohort studies on cardiovascular disease risk, 21 of which used definitions of metabolic health associated with metabolic syndrome[3]. Eleven definitions were derived from the National Cholesterol Education Program Adult Treatment Panel III (NCEP ATPIII)[4], five from the International Diabetes Federation (IDF)[5], and an additional five were based on the uniform international definition of metabolic syndrome（MetS) [6].

The NCEP ATP III criteria were used to define metabolic status. According to these criteria, unhealthy metabolic status（MetS) was defined as the presence of two or more relevant components.Based on BMI categories and metabolic status，we defined and distinguished four metabolic overweight/obesity phenotypes: metabolically healthy with normal weight (MHNW), metabolically unhealthy with normal weight (MUNW), metabolically healthy with overweight/obesity (MHO), and metabolically unhealthy with overweight/obesity (MUO). This classification approach is becoming increasingly common in studies aimed at highlighting various metabolic phenotypes of overweight/obesity and their impact on health outcomes.

For example, in the study by Hwi Seung Kim et al. [7], the NCEP ATP III criteria were used to define metabolic status and participants were divided into metabolically healthy non-obese group, metabolically unhealthy non-obese group, metabolically healthy obese group, and metabolically unhealthy obese group. In the study by Zinuo Yuan et al.[8], which also considered BMI categories and metabolic status, four metabolic overweight/obesity phenotypes were distinguished: MHNW, MUNW, MHO and MUO.

To enhance the communication of the core content and findings of our study, we considered implementing the following major changes:

**(1)Revised Title:** Association of the platelet-to-high-density lipoprotein cholesterol ratio with metabolic syndrome and metabolic overweight/obesity phenotypes: A study based on the Dryad database.

**(2)Revised Introduction:** Overweight/obesity is a significant risk factor for the development and progression of metabolic syndrome (MetS) [3,4]. MetS assessment relies on multiple indicators, including blood pressure, blood glucose, blood lipids, and other parameters [5,6].The metabolic status was assessed based on the presence or absence MetS [7]. Considering the association between BMI categories and metabolic status, individuals can be classified into one of four phenotypic groups: metabolically healthy normal weight (MHNW), metabolically unhealthy normal weight (MUNW), metabolically healthy overweight/obese (MHO), and metabolically unhealthy overweight/obese (MUO).

**(3)Methods and Results :**The methods and results section has been reanalyzed and rewritten. Please refer to the manuscript for further details.

Additionally, we identified some terminology confusion in the manuscript and made revisions.

We look forward to further feedback to refine this work.

References

1. Stefan N, Häring HU, Hu FB, Schulze MB. Metabolically healthy obesity: epidemiology, mechanisms, and clinical implications. Lancet Diabetes Endocrinol. 2013 Oct;1(2):152-62. doi: 10.1016/S2213-8587(13)70062-7.
2. Tsatsoulis A, Paschou SA. Metabolically Healthy Obesity: Criteria, Epidemiology, Controversies, and Consequences. Curr Obes Rep. 2020 Jun;9(2):109-120. doi: 10.1007/s13679-020-00375-0.
3. Yeh TL, Chen HH, Tsai SY, Lin CY, Liu SJ, Chien KL. The Relationship between Metabolically Healthy Obesity and the Risk of Cardiovascular Disease: A Systematic Review and Meta-Analysis. J Clin Med. 2019 Aug 15;8(8):1228.

doi: 10.3390/jcm8081228.

4. Detection EPo, Adults ToHBCi. Executive Summary of The Third Report of The National Cholesterol Education Program (NCEP) Expert Panel on Detection, Evaluation, And Treatment of High Blood Cholesterol In Adults (Adult Treatment Panel III). JAMA. 2001 May 16;285(19):2486-97. doi: 10.1001/jama.285.19.2486.

Alberti KGMM, Zimmet P, Shaw J. Metabolic syndrome--a new world-wide definition. A Consensus Statement from the International Diabetes Federation. Diabet Med. 2006 May;23(5):469-80. doi: 10.1111/j.1464-5491.2006.01858.x.

5. [Alberti KGMM, Eckel RH, Grundy SM, Zimmet PZ, Cleeman JI, Donato KA, et al. Harmonizing the metabolic syndrome: a joint interim statement of the International Diabetes Federation Task Force on Epidemiology and Prevention; National Heart, Lung, and Blood Institute; American Heart Association; World Heart Federation; International Atherosclerosis Society; and International Association for the Study of Obesity. Circulation. 2009 Oct 5;120(16):1640-5.](https://fanyi.medpeer.cn/article_library/abstract_library/abstractDetail?idm=abs20181213040304457132881129)

[doi: 10.1161/CIRCULATIONAHA.109.192644.](https://fanyi.medpeer.cn/article_library/abstract_library/abstractDetail?idm=abs20181213040304457132881129)

6. Kim HS, Lee J, Cho YK, Park JY, Lee WJ, Kim YJ, et al. Differential Effect of Metabolic Health and Obesity on Incident Heart Failure: A Nationwide Population-Based Cohort Study. Front Endocrinol (Lausanne). 2021 Feb 25;12:625083. doi: 10.3389/fendo.2021.625083.
7. Yuan Z, Cheng Y, Han J, Wang D, Dong H, Shi Y, et al. Association between metabolic overweight/obesity phenotypes and readmission risk in patients with lung cancer: A retrospective cohort study. EClinicalMedicine. 2022 Jul 22;51:101577. doi: 10.1016/j.eclinm.2022.101577.

**Comment 3: NAFLD is an old term. It is substituted with metabolic dysfunction associated with liver disease**

**Response:** We sincerely appreciate your review of our paper and your valuable comments. In 2020, the International Fatty Liver Expert Group recommended renaming non-alcoholic fatty liver disease (NAFLD) to metabolic dysfunction-associated fatty liver disease (MAFLD) to better reflect its relationship with metabolic health [1] . This amendment aims to highlight the pivotal role of metabolic dysfunction in the etiology of fatty liver disease. It is important to note that the term NAFLD has been replaced by MAFLD. **We have updated the relevant terminology in the manuscript.**

References

1. Eslam M, Newsome PN, Sarin SK, Anstee QM, Targher G, Romero-Gomez M, et al. A new definition for metabolic dysfunction-associated fatty liver disease: An international expert consensus statement. J Hepatol. 2020 Jul;73(1):202-209. doi: 10.1016/j.jhep.2020.03.039.

**Comment 4: The diagnostic criteria of body mass index is not according to the WHO criteria. Please add a reference of using the criteria of BMI that appeared in the article.**

**Response:** The World Health Organization's classical cutoff values for normal weight (18.5-24.9 kg/m²), overweight (25.0-29.9 kg/m²), and obesity (≥30.0 kg/m²) were used, while lower cutoff values were applied for the Asian population. A study by the Pan XF et al. published in 2021 in *The Lancet Diabetes & Endocrinology* noted that in the Chinese adult population, a BMI of less than 18.5 kg/m² indicates low weight, a BMI between 18.5 kg/m² and less than 24 kg/m² indicates normal weight, a BMI between 24 kg/m² and less than 28 kg/m² indicates overweight, and a BMI of 28 kg/m² or more indicates obesity [1]. We decided to use the Chinese obesity standard to classify BMI in our study [2] because this standard aligns more closely with the characteristics of the Chinese population and health risk assessment.

**Modified:**

In the Chinese adult population, a BMI of less than 18.5 kg/m² indicates low weight status, a BMI between 18.5 kg/m² and less than 24 kg/m² indicates normal weight, a BMI between 24 kg/m² and less than 28 kg/m² indicates overweight, and a BMI of 28 kg/m² or more indicates obesity ^[20]^. We decided to use the Chinese obesity standard to classify BMI in our study ^[21]^ because this standard aligns more closely with the characteristics of the Chinese population and health risk assessment.Patients were classified into BMI categories: normal weight (BMI <24 kg/m^2^), and overweight/obesity (BMI ≥24 kg/m^2^).

References

1. Pan XF, Wang L, and Pan A. Epidemiology and determinants of obesity in China. Lancet Diabetes Endocrinol. (2021) 9:373-392.

doi: 10.1016/S2213-8587(21)00045-0.

2. National Clinical Practice Guideline on Obesity Management Editorial Committee .National Clinical Practice Guideline on Obesity Management (2024 Edition).Chinese Circulation Journal. (2025)40: 6.

doi: 10.3969/j.issn.1000-3614.2025.01.002

**Responses to the Reviewer #1’s comments**

**Comment 1: The objective of the study is not stated clearly in the introduction. The authors should ameliorate this statement and show what is exactly known about PHR, obesity and metabolically unhealthy status.**

**Response:** Thank you for your constructive comments! We have made the following changes:

The platelet-to-high-density lipoprotein cholesterol ratio (PHR) has emerged as a novel marker of inflammatory response and metabolic disorders. PHR, a composite index combining platelet count and HDL-C levels, reflects both systemic inflammatory status and thrombotic tendency [14]. Recent studies have demonstrated strong associations between PHR and various chronic conditions, including hypertension, heart failure, and diabetes, highlighting its potential as a predictor of cardiometabolic diseases [15-17]. Obesity-induced chronic low-grade inflammation represents a key risk factor in the pathogenesis and progression of metabolic disorders [4]. A retrospective study [18] demonstrated that PHR can be utilized as a valid marker in obese patients to reliably predict the presence of type 2 diabetes. Alice Marra et al. [19] showed that five inflammatory indices derived from complete blood cell counts, including PHR, have good validity in predicting MetS in severely obese adults, providing clinicians with useful tools to assess obesity-related metabolic risk. While earlier research has examined the association between PHR and metabolic diseases such as hypertension and diabetes, there remains a paucity of information regarding the relationship between PHR and MetS and its defined overweight/obesity phenotypes. Consequently, the present study aims to analyze the relationship between PHR and MetS, as well as metabolic obesity/overweight phenotypes, with the goal of providing deeper insights for clinical practice.

**Comment 2: The scientific terminology is inappropriately reformulated thorough the text leading to confusions. For example, instead of saying “Classification of metabolic overweight /obesity phenotypes” use simple terms like: classification according to metabolic status and BMI.**

Response: Thank you for your valuable comments. We have made the following changes:

The individuals were categorized into four groups according to their BMI and metabolic status [24]:

(1) MHNW defined as BMI < 24 kg/m^2^ and unhealthy metabolic status;
(2) MHO defined as BMI ≥ 24 kg/m^2^and healthy metabolic status;
(3) MUNW defined as BMI < 24 kg/m^2^and unhealthy metabolic status;
(4) MUO defined as BMI ≥ 24 kg/m^2^and unhealthy metabolic status.

**Comment 3: The research methodology is misleading because of the choice of potential confounding factors for adjustments. Some confounders used by the authors cannot be consider as such because they were already used to classify the participants as MU or MH (Adult Treatment Panel III (ATP III) criteria (Blood pressure, diastolic and systolic, Fasting plasma glucose; High-density lipoprotein cholesterol (HDL-C), and Triglycerides) or to classify the participant as Obese and non-obese (BMI). So, they cannot be used as confounders. Hence,**

**In sections 3.2 and 3.3, as well as in the corresponding Tables 2 and 3, only two models should be kept: the unadjusted model and a model adjusted for these factors: gender, age, BMI, FMR. ALT, AST, UA, tobacco use, alcohol use and NAFLD.**

**The same comment can be done for Table 4 regarding the adjustment.**

**In subgroup analyses, do not include comorbidity conditions (hypertension, diabetes and NAFLD) because they are already associated to MU status.**

**Response:**Thank you for your in-depth review and valuable comments on our study. We greatly appreciate your feedback regarding the selection of potential confounders. Based on your suggestions, we have re-conducted the data analysis.

**Before modification：**

**Table 2 Associations between PHR and metabolic health status.**

| Variable | Molder1 | |  | Molder2 | |  | Molder3 | |
| --- | --- | --- | --- | --- | --- | --- | --- | --- |
|  | OR (95%CI) | *P*-value |  | OR (95%CI) | *P*-value |  | OR (95%CI) | *P*-value |
| PHR per 10 Units | 1.15 (1.13, 1.18) | <0.0001 |  | 1.16 (10.13, 1.19) | <0.0001 |  | 1.11 (1.07, 1.15) | <0.0001 |
| PHR tertile |  |  |  |  |  |  |  |  |
| T1(<164.55) | Ref. |  |  | Ref. |  |  | Ref. |  |
| T2(164.55-219.12) | 3.08 (2.37, 4.00) | <0.0001 |  | 3.02 (2.21, 4.11) | <0.0001 |  | 2.89 (1.80, 4.63) | <0.0001 |
| T3(>219.12) | 7.42 (5.42, 10.17) | <0.0001 |  | 8.18 (5.62, 11.92) | <0.0001 |  | 3.80 (2.20, 6.57) | <0.0001 |
| *P* for trend |  | <0.0001 |  |  | <0.0001 |  |  | <0.0001 |

Reference (Ref.); OR=Odds Ratio; CI= Confidence Interval; Molder1: unadjusted; Molder2: adjust for gender,age, BMI, and FMR. Molder 3: adjust for: gender,age, BMI, FMR, ALT, AST, UA, FBG, TC, TG, LDL-C, SBP, DBP, tobacco use, alcohol use, hypertension, diabetes, and NAFLD.

**Table 3. Associations between PHR and metabolically healthy overweight/obese phenotype.**

| Variable | Molder1 | |  | Molder2 | |  | Molder3 | |
| --- | --- | --- | --- | --- | --- | --- | --- | --- |
|  | β (95%CI) | *P*-value |  | β (95%CI) | *P*-value |  | β (95%CI) | *P*-value |
| MHNO | Ref. |  |  | Ref. |  |  | Ref. |  |
| MHO | 52.33 (39.82, 64.84) | <0.0001 |  | 58.98 (46.23, 71.73) | <0.0001 |  | 37.45 (24.32, 50.59) | <0.0001 |
| MUNO | 6.71 (-6.92, 20.34) | 0.33 |  | 12.55 (-0.89, 25.99) | 0.07 |  | 3.76 (-10.06, 17.59) | 0.59 |
| MUO | 60.50 (50.34, 70.66) | <0.0001 |  | 64.69 (53.80, 75.59) | <0.0001 |  | 33.02 (19.39, 46.66) | <0.0001 |
| *P* for trend |  | <0.001 |  |  | <0.001 |  |  | 0.005 |

Reference (Ref.); OR=Odds Ratio; CI= Confidence Interval; Molder1: unadjusted; Molder2: adjust for gender,age, BMI, and FMR. Molder 3: adjust for: gender,age, BMI, FMR, ALT, AST, UA, FBG, TC, TG, LDL-C, SBP, DBP, tobacco use, alcohol use, hypertension, diabetes, and NAFLD.

**Table 4. Threshold effect analysis of PHR on MU using piecewise linear regression.**

| Inflection point of PHR (U) | OR（95%C)* | p value |
| --- | --- | --- |
| <185.09 | 1.49(1.27, 1.76) | <0.0001 |
| ≥185.09 | 1.11 (1.01, 1.23) | 0.04 |
| Logarithmic likelihood ratio test *P* value |  | 0.01 |

*PHR per 20 units; adjust for: gender, age, BMI, FMR, ALT, AST, UA, FBG, TC, TG, LDL-C, SBP, DBP, tobacco use, alcohol use, hypertension, diabetes, and NAFLD.

**Figure 3. Forest plot of stratified analyses for the association between PHR and MU.**

**After modification:**

**Table 2 Associations between PHR and MetS.**

| Variable | Molder1 | |  | Molder2 | |  | Molder3 | |
| --- | --- | --- | --- | --- | --- | --- | --- | --- |
|  | OR (95%CI) | *P*-value |  | OR (95%CI) | *P*-value |  | OR (95%CI) | *P*-value |
| PHR per 10 Units | 1.15 (1.13, 1.18) | <0.0001 |  | 1.16 (10.13, 1.19) | <0.0001 |  | 1.29 (1.24, 1.35) | <0.0001 |
| PHR tertile |  |  |  |  |  |  |  |  |
| T1(<164.55) | Ref. |  |  | Ref. |  |  | Ref. |  |
| T2(164.55-219.12) | 3.08 (2.37, 4.00) | <0.0001 |  | 3.02 (2.21, 4.11) | <0.0001 |  | 4.04 (2.85, 5.73) | <0.0001 |
| T3(>219.12) | 7.42 (5.42, 10.17) | <0.0001 |  | 8.18 (5.62, 11.92) | <0.0001 |  | 16.10 (9.75, 26.60) | <0.0001 |
| *P* for trend |  | <0.0001 |  |  | <0.0001 |  |  | <0.0001 |

Reference (Ref.); OR=Odds Ratio; CI= Confidence Interval; Molder1: unadjusted; Molder2: adjust for gender,age, BMI, and FMR. Molder 3: adjust for: gender,age, BMI, FMR, ALT, AST, UA, tobacco use, and alcohol use.

**Table 3. Threshold effect analysis of PHR on probability of MetS using piecewise linear regression.**

| Inflection point of PHR (U) | OR（95%CI)* | p value |
| --- | --- | --- |
| <273.53 | 1.17(1.13, 1.21) | <0.0001 |
| ≥273.53 | 1.05 (0.97, 1.14) | 0.25 |
| Logarithmic likelihood ratio test *P* value |  | 0.047 |

*PHR per 10 units; adjust for: gender,age, FMR, ALT, AST, UA, tobacco use, alcohol use.

**Table 4. Multivariate logistic analysis associations between PHR and metabolically overweight/obese phenotype.**

| Variable | Molder1 | |  | Molder2 | |  | Molder3 | |
| --- | --- | --- | --- | --- | --- | --- | --- | --- |
|  | OR (95%CI) | *P*-value |  | OR (95%CI) | *P*-value |  | OR (95%CI) | *P*-value |
| **Incidence of MHO** |  |  |  |  |  |  |  |  |
| PHR per 10 Units | 1.16(1.12, 1.19) | <0.0001 |  | 1.17 (1.13, 1.22) | <0.0001 |  | 1.15(1.11, 1.19) | <0.0001 |
| PHR tertile |  |  |  |  |  |  |  |  |
| T1(<164.55) | Ref. |  |  | Ref. |  |  | Ref. |  |
| T2(164.55-219.12) | 3.26 (2.14, 4.98) | <0.0001 |  | 3.59 (2.24, 5.75) | <0.0001 |  | 3.28 (2.01, 5.34) | <0.0001 |
| T3(>219.12) | 5.70 (3.58, 9.07) | <0.0001 |  | 7.80 (4.59, 13.27) | <0.0001 |  | 6.25 (3.62, 10.78) | <0.0001 |
| **Incidence of MUNW** |  |  |  |  |  |  |  |  |
| PHR per 10 Units | 1.03(0.99, 1.06) | 0.17 |  | 1.02 (0.98, 1.07) | 0.315 |  | 1.01 (0.96, 1.05) | 0.73 |
| PHR tertile |  |  |  |  |  |  |  |  |
| T1(<164.55) | Ref. |  |  | Ref. |  |  | Ref. |  |
| T2(164.55-219.12) | 1.55 (1.01, 2.38) | 0.04 |  | 1.32 (0.79, 2.19) | 0.29 |  | 1.24 (0.74, 2.08) | 0.42 |
| T3(>219.12) | 0.95 (0.54, 1.68) | 0.86 |  | 1.07 (0.56, 2.07) | 0.83 |  | 0.93 (0.48, 1.81) | 0.83 |
| **Incidence of MUO** |  |  |  |  |  |  |  |  |
| PHR per 10 Units | 1.17(1.14, 1.20) | <0.0001 |  | 1.18(1.14, 1.22) | <0.0001 |  | 1.16(1.12, 1.20) | <0.0001 |
| PHR tertile |  |  |  |  |  |  |  |  |
| T1(<164.55) | Ref. |  |  | Ref. |  |  | Ref. |  |
| T2(164.55-219.12) | 3.95 (2.80, 5.56) | <0.0001 |  | 3.45 (2.16, 5.49) | <0.0001 |  | 3.10(1.92, 5.02) | <0.0001 |
| T3(>219.12) | 7.87 (5.30, 11.67) | <0.0001 |  | 9.08 (5.29, 15.59) | <0.0001 |  | 7.15 (4.14, 12.36) | <0.0001 |

Reference (Ref.);OR=Odds Ratio; CI= Confidence Interval; Molder1: unadjusted; Molder2: adjust for gender,age, and FMR. Molder 3: adjust for: gender,age, FMR, ALT, AST, UA, tobacco use, and alcohol use.

**Figure 4. Forest plot of stratified analyses for the association between PHR and MetS.** OR and 95%CI are shown for each 10-unit increase in PHR, stratified by demographic and clinical characteristics. Analyses were adjusted for potential confounders except for the stratification variable. The vertical dashed line represents OR = 1.0. *P*-interaction values indicate the significance of effect modification between subgroups.

**Comment 4: The comparison of PHR across the 4 phenotypes (Figure 1) should be done by ANOVA test because the Kruskal-Wallis test is a non-parametric test and the sample size is considerable.**

**Response:**Thank you for your review of our paper and your valuable comments. In response to your suggestion that the ANOVA test should be used to compare PHR among the four phenotypes (MHNW, MHO, MUNW, MUO), we would like to provide further clarification regarding our choice of analysis.

We performed multiple normality tests (e.g., Anderson-Darling, Cramer-von Mises, Lilliefors, Pearson chi-square, and Shapiro-Francia tests), all of which yielded p-values of less than 0.000001. The null hypothesis was strongly rejected, indicating that the data did not conform to a normal distribution. This finding suggests that we should not assume a normal distribution for subsequent statistical analyses (See Fig.S1）.

**Fig.S1 Results of multiple normality tests.**

ANOVA is a parametric test that assumes the data are normally distributed and that the variances among groups are equal. Since our data did not meet these assumptions, using ANOVA could have resulted in inaccurate and misleading results.

The Kruskal-Wallis test is a nonparametric test suitable for comparing the medians of three or more independent samples, particularly when the data do not follow a normal distribution. Given that our data deviated significantly from normality, the Kruskal-Wallis test was more effective in handling these data, ensuring the validity and reliability of the analysis results.

Based on the analysis above, we decided to use the Kruskal-Wallis test instead of ANOVA for comparing PHR among different phenotypes. This choice was made to ensure that our statistical analysis aligned with the actual distribution characteristics of the data, thereby increasing confidence in the results.

Of course, we also conducted an ANOVA analysis based on your suggestions, and the results are presented in Fig.S2. Both analytical methods demonstrated consistent differences between the groups.

**Fig S2.** **PHR levels in different metabolically obesity phenotypes.** ****P* < 0.001 by ANOVA test.

We are very grateful for your attention and support regarding our study. If you have any further questions or suggestions, we would be happy to discuss them.

**Comment 5: Table 1: please mention in the title “by PHR tertiles”**

**Response:** Thank you for your valuable comments. It has been added to the article:

Table 1. Clinical characteristics of the study participants by PHR tertiles.

**Comment 6: Put the section 3.3 (Smooth curve fitting and threshold effect analysis) after the section 3. 2**

**Response:** Thank you for your valuable comments. The data were reanalyzed, and the manuscript was substantially revised:

**3.2 Association between PHR and MetS.**

Table 2 demonstrates the associations between PHR and MetS across three statistical models. When analyzed as a continuous variable, each 10-unit increase in PHR was associated with elevated risks of MetS in all models (Model 1: OR=1.15, 95% CI: 1.13-1.18; Model 2: OR=1.16, 95% CI: 1.13-1.19; Model 3: OR=1.29, 95% CI: 1.24-1.35). In the categorical analysis using PHR tertiles (T1: <164.55, T2: 164.55-219.12, T3: >219.12), compared to the lowest tertile, both middle and highest tertiles showed significantly increased risks. The T2 group demonstrated approximately three-fold increased risks across all models (Model 3: OR=4.04, 95% CI: 2.85-5.73), while the highest tertile showed more pronounced associations (Model 1: OR=7.42, 95% CI: 5.42-10.17; Model 2: OR=8.18, 95% CI: 5.62-11.92; Model 3: OR=16.10, 95% CI: 9.75-26.60). Trend tests were significant across all models (P<0.0001), confirming a robust dose-dependent relationship between PHR and MetS, which persisted after comprehensive adjustment for potential confounders.

Figure 1 illustrates a nonlinear relationship between PHR and MetS after adjustment for potential confounders. Threshold effect analysis identified an inflection point at PHR = 273.53 (Table 3). Below this threshold, PHR demonstrated a strong positive association with the risk of MetS, characterized by a steep upward trend. Above 273.53, the relationship plateaued, showing a markedly attenuated rate of increase in MetS risk with further elevations in PHR.

**Table 2 Associations between PHR and MetS.**

| Variable | Model 1 | |  | Model 2 | |  | Model 3 | |
| --- | --- | --- | --- | --- | --- | --- | --- | --- |
|  | OR (95%CI) | *P*-value |  | OR (95%CI) | *P*-value |  | OR (95%CI) | *P*-value |
| PHR per 10 Units | 1.15 (1.13, 1.18) | <0.0001 |  | 1.16 (10.13, 1.19) | <0.0001 |  | 1.29 (1.24, 1.35) | <0.0001 |
| PHR tertile |  |  |  |  |  |  |  |  |
| T1(<164.55) | Ref. |  |  | Ref. |  |  | Ref. |  |
| T2(164.55-219.12) | 3.08 (2.37, 4.00) | <0.0001 |  | 3.02 (2.21, 4.11) | <0.0001 |  | 4.04 (2.85, 5.73) | <0.0001 |
| T3(>219.12) | 7.42 (5.42, 10.17) | <0.0001 |  | 8.18 (5.62, 11.92) | <0.0001 |  | 16.10 (9.75, 26.60) | <0.0001 |
| *P* for trend |  | <0.0001 |  |  | <0.0001 |  |  | <0.0001 |

Reference (Ref.); OR=Odds Ratio; CI= Confidence Interval; Model 1: unadjusted; Model 2: adjust for gender,age, BMI, and FMR. Model 3: adjust for: gender,age, BMI, FMR, ALT, AST, UA, tobacco use, and alcohol use.

**Table 3. Threshold effect analysis of PHR on incidence of MetS.**

| Inflection point of PHR (U) | OR（95%CI)* | p value |
| --- | --- | --- |
| <273.53 | 1.17(1.13, 1.21) | <0.0001 |
| ≥273.53 | 1.05 (0.97, 1.14) | 0.25 |
| Logarithmic likelihood ratio test *P* value |  | 0.047 |

*PHR per 10 units; adjust for: gender,age, FMR, ALT, AST, UA, tobacco use, and alcohol use.

**3.3 Association between PHR and metabolic overweight/obese phenotypes**

**Figure 2** demonstrate the distribution of PHR across different metabolically overweight/obese phenotypes. The MUO group exhibited the highest PHR level (221.05), while the MHNW group showed the lowest (160.55). The results of the logistic regression analysis are presented in **Table 4**. The findings indicate that for every 10-unit increase in PHR, the likelihood of MHO occurrence significantly increases (Model 1:OR=1.16, 95%CI=1.12-1.19; Model 2:OR=1.17, 95%CI=1.13-1.22; Model 3:OR=1.15, 95%CI=1.11-1.19). In the quantile analysis of PHR, the OR for the T2 and T3 groups were 3.26 (95% CI: 2.14 - 4.98) and 5.70 (95% CI: 3.58 - 9.07), respectively, indicating a significant increase in risk. Conversely, the association between PHR and the occurrence of MUNW was not significant, with P-values for all models exceeding 0.05. In contrast, the association between PHR and MUO was significant.For each 10-unit increase in PHR, the risk of MUO occurrence significantly increased, with an OR of 1.17 (95% CI: 1.14 - 1.20). The OR for the T2 and T3 groups were 3.95 (95% CI: 2.80 - 5.56) and 7.87 (95% CI: 5.30 - 11.67), respectively.These results indicate a significant positive association between PHR and the occurrence of both MHO and MUO, while the association with MUNW remains unclear.

Using generalized additive models and restricted cubic spline analysis, we further explored the correlation between PHR and the risk of MHO or MUNO. **Figure 3** illustrates a nonlinear relationship between PHR and incidence of MHO (Fig.3A) and MUO (Fig.3B) after adjustment for potential confounders. We identified inflection points at 166.67 for MHO and 236.36 for MUO, respectively. When PHR was below 166.67, the risk of MHO increased by 28% (P < 0.0001). When PHR was ≥ 166.67, the increase in MHO risk significantly slowed (P < 0.0001). When PHR was below 236.36, the risk of MUO increased by 21% (P < 0.0001). When PHR was ≥ 236.36, it was no longer significantly associated with MUO (P = 0.159). Refer to Table 5 for detailed information.

**Table 4. Multivariate logistic analysis associations between PHR and metabolic overweight/obese phenotypes.**

| Variable | Model1 | |  | Model 2 | |  | Model 3 | |
| --- | --- | --- | --- | --- | --- | --- | --- | --- |
|  | OR (95%CI) | *P*-value |  | OR (95%CI) | *P*-value |  | OR (95%CI) | *P*-value |
| **Incidence of MHO** |  |  |  |  |  |  |  |  |
| PHR per 10 Units | 1.16(1.12, 1.19) | <0.0001 |  | 1.17 (1.13, 1.22) | <0.0001 |  | 1.15(1.11, 1.19) | <0.0001 |
| PHR tertile |  |  |  |  |  |  |  |  |
| T1(<164.55) | Ref. |  |  | Ref. |  |  | Ref. |  |
| T2(164.55-219.12) | 3.26 (2.14, 4.98) | <0.0001 |  | 3.59 (2.24, 5.75) | <0.0001 |  | 3.28 (2.01, 5.34) | <0.0001 |
| T3(>219.12) | 5.70 (3.58, 9.07) | <0.0001 |  | 7.80 (4.59, 13.27) | <0.0001 |  | 6.25 (3.62, 10.78) | <0.0001 |
| **Incidence of MUNW** |  |  |  |  |  |  |  |  |
| PHR per 10 Units | 1.03(0.99, 1.06) | 0.17 |  | 1.02 (0.98, 1.07) | 0.315 |  | 1.01 (0.96, 1.05) | 0.73 |
| PHR tertile |  |  |  |  |  |  |  |  |
| T1(<164.55) | Ref. |  |  | Ref. |  |  | Ref. |  |
| T2(164.55-219.12) | 1.55 (1.01, 2.38) | 0.04 |  | 1.32 (0.79, 2.19) | 0.29 |  | 1.24 (0.74, 2.08) | 0.42 |
| T3(>219.12) | 0.95 (0.54, 1.68) | 0.86 |  | 1.07 (0.56, 2.07) | 0.83 |  | 0.93 (0.48, 1.81) | 0.83 |
| **Incidence of MUO** |  |  |  |  |  |  |  |  |
| PHR per 10 Units | 1.17(1.14, 1.20) | <0.0001 |  | 1.18(1.14, 1.22) | <0.0001 |  | 1.16(1.12, 1.20) | <0.0001 |
| PHR tertile |  |  |  |  |  |  |  |  |
| T1(<164.55) | Ref. |  |  | Ref. |  |  | Ref. |  |
| T2(164.55-219.12) | 3.95 (2.80, 5.56) | <0.0001 |  | 3.45 (2.16, 5.49) | <0.0001 |  | 3.10(1.92, 5.02) | <0.0001 |
| T3(>219.12) | 7.87 (5.30, 11.67) | <0.0001 |  | 9.08 (5.29, 15.59) | <0.0001 |  | 7.15 (4.14, 12.36) | <0.0001 |

Reference (Ref.);OR=Odds Ratio; CI= Confidence Interval; Model 1: unadjusted; Model 2: adjust for gender,age, and FMR. Model 3: adjust for: gender,age, FMR, ALT, AST, UA, tobacco use, and alcohol use.

**Table 5. Threshold effect analysis of PHR on incidence of MHO and MUNO.**

| Inflection point of PHR (U) | OR（95%CI)* | p value |
| --- | --- | --- |
| **MHO** |  |  |
| <166.67 | 1.28(1.14, 1.44) | <0.0001 |
| ≥166.67 | 1.12 (1.06, 1.18) | <0.0001 |
| Logarithmic likelihood ratio test *P* value |  | 0.056 |
| **MUO** |  |  |
| <236.36 | 1.21 (1.13, 1.28) | <0.0001 |
| ≥236.36 | 1.07 (0.97, 1.18) | 0.159 |
| Logarithmic likelihood ratio test *P* value |  | 0.094 |

*PHR per 10 units; adjust for: gender,age, FMR, ALT, AST, UA, tobacco use, and alcohol use.

**Comment 7: Use the same abbreviations: sometimes we can read MHNW and sometimes MHNO.**

**Response:** Thank you for your valuable feedback regarding the abbreviations. Based on BMI categories and metabolic status，we defined and distinguished four metabolic overweight/obesity phenotypes: metabolically healthy with normal weight (MHNW), metabolically unhealthy with normal weight (MUNW), metabolically healthy with overweight or obesity (MHO), and metabolically unhealthy with overweight or obesity (MUO).

I have reviewed the manuscript and ensured consistency by using the same abbreviation throughout. Your attention to detail is greatly appreciated.

**Comment 8: In section 3.3 and table 4 title, mention MU “probability”**

Response: Thank you for your detailed review and valuable comments. We've made changes. For example:Table 3. Threshold effect analysis of PHR on incidence of MetS. Table 5. Threshold effect analysis of PHR on incidence of MHO and MUNO.

**Comment 9: In section 3.4, figure 3 is not mentioned.**

**Response:** Thank you for your constructive comments! We've added it.

**Responses to the Reviewer #2’s comments**

**Comment 1:This retrospective cross-sectional study utilized data from the Dryad digital repository (doi : 10.5061/dryad.7d7wm3809). This design poses a problem: is it a cross-sectional analytical study? Or a retrospective case-control study?A cross-sectional study is, par excellence, a prevalence study, taking into account that the study population included people who underwent full health examinations at Wuhan Union Medical College Hospital between January 2020 and November 2021. Clearly, the survey was not carried out in the community, but the data was hospital-based. What did you do to minimise this bias in relation to a cross-sectional design?You just mentioned the study population. Did you draw a sample from this population, and if so, how did you do it?
Response:** Thank you for reviewing our paper and for your valuable comments. Regarding the study design you mentioned, we would like to provide the following explanation:

This study was a retrospective cross-sectional analysis utilizing data from the Dryad database (DOI: 10.5061/dryad.7d7wm3809). It focused on a population that underwent medical examinations at Wuhan Union Hospital from January 2020 to November 2021. During this period, body composition analysis, liver ultrasound examinations, and related information collection were conducted, aligning with the characteristics of cross-sectional studies that observe and collect data from a population within a specific timeframe.

It is important to note that this is not a retrospective case-control study. In case-control studies, subjects are first divided into case and control groups based on disease status, and then potential risk factors are identified. In contrast, this study investigated the prevalence of fatty liver and NAFLD, along with related factors (such as fat-to-muscle ratio), across the overall population.

This study did not sample from the population; instead, we consecutively included individuals aged 40 to 79 years who voluntarily underwent body composition analysis and liver ultrasound as part of their health examination. This method of continuous enrollment helps avoid potential selection bias during the sampling process, ensuring that the study subjects represent a broad range of eligible individuals during this time period and enhancing the representativeness of the study results. Ultimately, of the initial 1,830 individuals, 1,592 participants were included for subsequent analyses after applying the exclusion criteria.

The single-center retrospective design may have introduced selection bias, potentially limiting the generalizability of our findings. This represents one of the key limitations of the study.

**Comment 2: 134 EmpowerStats (version 4.2). Continuous variables are presented as mean ± standard deviation, and categorical variables as numbers (percentages). And what did you do for the variables whose distribution was not normal?
Respone:** We assessed the distribution of continuous variables using histograms, Q-Q plots, and the Shapiro-Wilk test. For variables that significantly deviated from normality, we presented the data as median and interquartile range (IQR) instead of mean ± standard deviation. Non-parametric tests such as the Mann-Whitney U test or Kruskal-Wallis test were used for group comparisons, as appropriate. These methods were chosen because they are more robust to deviations from normality and provide a more accurate representation of the data's central tendency and variability.We have made the following changes in ‘2.6 Statistical Analysis’:

Summaries of continuous variables are presented as means and standard deviations unless skewed and then presented as medians and interquartile ranges(IQR).

In addition, we re-analyzed the baseline data presented in Table 1, with the modified sections highlighted in red.

**Table 1. Clinical characteristics of the study participants by PHR tertiles.**

| Characters | Total | T1 group | T2 group | T3 group | *P*-value |
| --- | --- | --- | --- | --- | --- |
| PHR | (47.52-694.59) | <164.55 | 164.55-219.12 | >219.12 |  |
| *n* | 1592 | 531 | 530 | 531 |  |
| Age group, years, n(%) |  |  |  |  | <0.001 |
| 40-49 | 354 (22.24) | 91 (17.14) | 115 (21.70) | 148 (27.87) |  |
| 50-59 | 709 (44.54) | 198 (37.29) | 259 (48.87) | 252 (47.46) |  |
| 60-69 | 360 (22.61) | 163 (30.70) | 104 (19.62) | 93 (17.51) |  |
| 70-79 | 169 (10.62) | 79 (14.88) | 52 (9.81) | 38 (7.16) |  |
| Gender, n(%) |  |  |  |  | <0.001 |
| Female | 444 (27.89%) | 206 (38.79%) | 138 (26.04%) | 100 (18.83) |  |
| Male | 1148 (72.11%) | 325 (61.21%) | 392 (73.96%) | 431 (81.17) |  |
| BMI, kg/m^2^ | 25.10 (23.30, 27.10) | 24.30 (22.60, 26.50) | 25.10 (23.60, 27.30) | 25.60 (23.80, 27.60) | <0.001 |
| FMR | 0.37 (0.31, 0.45) | 0.38 (0.31, 0.48) | 0.37 (0.32, 0.46) | 0.37 (0.32, 0.43) | 0.102 |
| Tobacco use, n (%) |  |  |  |  | <0.001 |
| No | 1054 (66.21) | 401 (75.52) | 343 (64.72) | 310 (58.38) |  |
| Yes | 538 (33.79) | 130 (24.48) | 187 (35.28) | 221 (41.62) |  |
| Alcohol use, n (%) |  |  |  |  | 0.004 |
| No | 1072 (67.34) | 386 (72.69) | 349 (65.85) | 337 (63.47) |  |
| Yes | 520 (32.66) | 145 (27.31) | 181 (34.15) | 194 (36.53) |  |
| Hypertension, n (%) |  |  |  |  | 0.003 |
| No | 649 (40.77) | 230 (43.31) | 234 (44.15) | 185 (34.84) |  |
| Yes | 943 (59.23) | 301 (56.69) | 296 (55.85) | 346 (65.16) |  |
| Diabetes, n (%) |  |  |  |  | <0.001 |
| No | 1094 (68.72) | 390 (73.45) | 372 (70.19) | 332 (62.52) |  |
| Yes | 498 (31.28) | 141 (26.55) | 158 (29.81) | 199 (37.48) |  |
| MAFLD, n (%) |  |  |  |  | <0.001 |
| No | 619 (38.88) | 271 (51.04) | 196 (36.98) | 152 (28.63) |  |
| Yes | 973 (61.12) | 260 (48.96) | 334 (63.02) | 379 (71.37) |  |
| MetS |  |  |  |  | <0.001 |
| No | 453 (28.45) | 263 (49.53) | 128 (24.15) | 62 (11.68) |  |
| Yes | 1139 (71.55) | 268 (50.47) | 402 (75.85) | 469 (88.32) |  |
| Metabolic overweight/obesity phenotypes | |  |  |  | <0.001 |
| MHNW | 259 (16.27) | 158 (29.76) | 63 (11.89) | 38 (7.16) |  |
| MHO | 268 (16.83) | 73 (13.75) | 95 (17.92) | 100 (18.83) |  |
| MUNW | 194 (12.19) | 105 (19.77) | 65 (12.26) | 24 (4.52) |  |
| MUO | 871 (54.71) | 195 (36.72) | 307 (57.92) | 369 (69.49) |  |
| PLT, 10^9^/L | 207 (175, 241) | 174 (149, 202) | 206 (180, 233) | 241 (213, 280) | <0.001 |
| ALT, U/L | 21 (15, 30) | 19 (14, 26) | 22 (15, 30) | 24 (17, 36) | <0.001 |
| AST, U/L | 21 (17, 26) | 21 (17, 25) | 21 (17, 26) | 21 (17, 27) | 0.593 |
| FBG, mmol/L | 5.10 (4.70, 5.70) | 5.00 (4.60, 5.60) | 5.15 (4.70, 5.76) | 5.20 (4.80, 5.90) | <0.001 |
| UA, µmol/L | 363 (301, 426) | 335 (273, 396) | 369 (307, 435) | 378 (317, 445) | <0.001 |
| TC, mmol/L | 4.44 (3.73, 5.14) | 4.37 (3.62, 5.09) | 4.51 (3.80, 5.16) | 4.43 (3.75, 5.16) | 0.107 |
| TG, mmol/L | 1.42 (0.98, 2.18) | 1.03 (0.78, 1.48) | 1.49 (1.06, 2.15) | 1.89 (1.29, 2.90) | <0.001 |
| HDL-C, mmol/L | 1.07 (0.89, 1.28) | 1.33 (1.15, 1.54) | 1.07 (0.94, 1.20) | 0.88 (0.76, 1.01) | <0.001 |
| LDL-C, mmol/L | 2.65 (2.02, 3.22) | 2.54 (1.89, 3.09) | 2.73 (2.09, 3.30) | 2.67 (2.12, 3.26) | <0.001 |
| SBP, mmHg | 130 (120, 140) | 128 (120, 140) | 130 (120, 140) | 130 (121, 141) | 0.032 |
| DBP, mmHg | 80 (74, 89) | 80 (73, 88) | 81 (74, 90) | 81 (76, 90) | <0.001 |

Continuous variables are presented as Median (IQR). Categorical variables are presented as n (%). Univariate logistic regression models were used for continuous and categorical variables. ALT= alanine aminotransferase, AST= aspartate aminotransferase, BMI= body mass index, DBP= diastolic blood pressure, FBG= fasting blood glucose, FMR= fat-to-muscle ratio, HDL-C= high-density lipoprotein-cholesterol, LDL-C= low-density lipoprotein-cholesterol, MetS= metabolic syndrome, MHNW= metabolically healthy with normal weight, MHO= metabolically healthy with overweight/obesity, MUNW= metabolically unhealthy with normal weight, MUO= metabolically unhealthy with overweight/obesity, MAFLD=metabolic associated fatty liver disease, PLT= platelet, SBP= systolic blood pressure, TC= total cholesterol, TG= triglyceride, UA= uric acid.

**Comment 3:** The independent association between PHR and metabolic health status (including metabolically healthy overweight/obesity phenotypes) was assessed using logistic regression analysis. Logistic regression involves testing a regression model in which the dependent variable is dichotomous (coded 0-1). In this study, can you clarify what the dependent variable is and how it works?

**Respone:** Thank you for your interest in our study and for your insightful questions. Following a thorough investigation, it was determined that the software operation method employed in this particular section had been utilized in an erroneous manner. To ascertain the most accurate conclusions, consultations were undertaken with statistical experts, and a re-analysis of the data was conducted. The ensuing findings are outlined below:

**Table 4. Multivariate logistic analysis associations between PHR and metabolically healthy overweight/obese phenotype.**

| Variable | Molder1 | |  | Molder2 | |  | Molder3 | |
| --- | --- | --- | --- | --- | --- | --- | --- | --- |
|  | OR (95%CI) | *P*-value |  | OR (95%CI) | *P*-value |  | OR (95%CI) | *P*-value |
| **Incidence of MHO** |  |  |  |  |  |  |  |  |
| PHR per 10 Units | 1.16(1.12, 1.19) | <0.0001 |  | 1.17 (1.13, 1.22) | <0.0001 |  | 1.15(1.11, 1.19) | <0.0001 |
| PHR tertile |  |  |  |  |  |  |  |  |
| T1(<164.55) | Ref. |  |  | Ref. |  |  | Ref. |  |
| T2(164.55-219.12) | 3.26 (2.14, 4.98) | <0.0001 |  | 3.59 (2.24, 5.75) | <0.0001 |  | 3.28 (2.01, 5.34) | <0.0001 |
| T3(>219.12) | 5.70 (3.58, 9.07) | <0.0001 |  | 7.80 (4.59, 13.27) | <0.0001 |  | 6.25 (3.62, 10.78) | <0.0001 |
| **Incidence of MUNW** |  |  |  |  |  |  |  |  |
| PHR per 10 Units | 1.03(0.99, 1.06) | 0.17 |  | 1.02 (0.98, 1.07) | 0.315 |  | 1.01 (0.96, 1.05) | 0.73 |
| PHR tertile |  |  |  |  |  |  |  |  |
| T1(<164.55) | Ref. |  |  | Ref. |  |  | Ref. |  |
| T2(164.55-219.12) | 1.55 (1.01, 2.38) | 0.04 |  | 1.32 (0.79, 2.19) | 0.29 |  | 1.24 (0.74, 2.08) | 0.42 |
| T3(>219.12) | 0.95 (0.54, 1.68) | 0.86 |  | 1.07 (0.56, 2.07) | 0.83 |  | 0.93 (0.48, 1.81) | 0.83 |
| **Incidence of MUO** |  |  |  |  |  |  |  |  |
| PHR per 10 Units | 1.17(1.14, 1.20) | <0.0001 |  | 1.18(1.14, 1.22) | <0.0001 |  | 1.16(1.12, 1.20) | <0.0001 |
| PHR tertile |  |  |  |  |  |  |  |  |
| T1(<164.55) | Ref. |  |  | Ref. |  |  | Ref. |  |
| T2(164.55-219.12) | 3.95 (2.80, 5.56) | <0.0001 |  | 3.45 (2.16, 5.49) | <0.0001 |  | 3.10(1.92, 5.02) | <0.0001 |
| T3(>219.12) | 7.87 (5.30, 11.67) | <0.0001 |  | 9.08 (5.29, 15.59) | <0.0001 |  | 7.15 (4.14, 12.36) | <0.0001 |

Reference (Ref.);OR=Odds Ratio; CI= Confidence Interval; Molder1: unadjusted; Molder2: adjust for gender,age, and FMR. Molder 3: adjust for: gender,age, FMR, ALT, AST, UA, tobacco use, and alcohol use.

The results of the multivariate logistic regression analysis are presented in Table 4. The findings indicate that for every 10-unit increase in PHR, the likelihood of MHO occurrence significantly increases (Model 1:OR=1.16, 95%CI=1.12-1.19; Model 2:OR=1.17, 95%CI=1.13-1.22; Model 3:OR=1.15, 95%CI=1.11-1.19). In the quantile analysis of PHR, the OR for the T2 and T3 groups were 3.26 (95% CI: 2.14 - 4.98) and 5.70 (95% CI: 3.58 - 9.07), respectively, indicating a significant increase in risk. Conversely, the association between PHR and the occurrence of MUNW was not significant, with P-values for all models exceeding 0.05.

We believe that these modifications will enhance the scientific validity and clinical relevance of the article. Thank you once again for your valuable comments.
